# Factors and a configurational pathway associated with lower turnover intention among high-level medical professionals in county-level hospitals in China: a multicenter cross-sectional study

**DOI:** 10.3389/fpubh.2026.1917115

**Published:** 2026-09-08

**Authors:** Shuxin Dong, Qiuyuan Tai, Yiming Yao, Junlong Shen, Xuemei Huang

**Affiliations:** 1 School of Health Economics and Management, Nanjing University of Chinese Medicine, Nanjing, China; 2 Personnel Section, Nanjing Lishui People's Hospital, Zhongda Hospital Lishui Branch, Southeast University, Nanjing, China

**Keywords:** confirmatory factor analysis, county-level hospitals, Delphi method, exploratory factor analysis, fuzzy-set qualitative comparative analysis, high-level medical professionals, lower turnover intention

## Abstract

**Objective:**

This study developed a context-specific framework of perceived conditions related to lower turnover intention among high-level medical professionals in county-level hospitals and examined the configurational pattern associated with a high retention-oriented score.

**Methods:**

A multicenter cross-sectional study combined three Delphi rounds, psychometric evaluation, and fuzzy-set qualitative comparative analysis (fsQCA). The Delphi process progressed from 33 items in round 1–37 in round 2 and 34 assessed in round 3, with 33 retained for the survey. From July 2024 to February 2025, online and paper questionnaires were distributed in 10 county-level public hospitals in Jiangsu Province. Of 635 questionnaires distributed, 611 were returned and 579 were valid. The sample was randomly split for exploratory factor analysis (EFA; *n* = 289) and confirmatory factor analysis (CFA; *n* = 290). fsQCA used the full sample, a frequency threshold of 10, consistency of 0.85, and proportional reduction in inconsistency (PRI) of 0.70.

**Results:**

Thirty experts participated in Delphi round 1 and 29 in rounds 2 and 3 after one expert retired. The final 31 condition items showed high internal consistency (alpha = 0.971), suggesting both reliability and possible redundancy. Parallel analysis in the EFA half-sample suggested two factors, whereas a theory-constrained five-factor solution remained interpretable. In the CFA half-sample, the correlated five-factor model fit was marginal (Satorra-Bentler chi-square = 1350.48, df = 424, CFI = 0.903, TLI = 0.893, RMSEA = 0.087, 90% CI 0.082–0.092, SRMR = 0.042) and was similar to the second-order and bifactor models. No condition met the 0.90 necessity threshold. The corrected truth-table analysis identified one high-score configuration, PD^*^IES^*^CIS^*^OIE (consistency = 0.953; PRI = 0.917; coverage = 0.631). PD and CIS were core present conditions; IES and OIE were peripheral present conditions; IEPS was not included.

**Conclusion:**

A single sufficient configuration combined favorable perceptions of personal development, institutional/environmental support, compensation/incentive support, and organizational/interpersonal empowerment. The result does not support multiple substitutable pathways. Because the outcome was a retention-oriented score derived from a turnover-intention measure, the findings concern lower turnover intention rather than directly observed retention. The cross-sectional, individual-level results indicate associations and should not be interpreted causally or at the hospital level.

## 1 Introduction

As the Healthy China strategy, the tiered diagnosis and treatment system, and county-level medical consortiums continue to advance, county-level hospitals have assumed an increasingly important role in China's health system (1–4). They are the highest-level hospitals in the county-based three-tier network and provide comprehensive services for local populations while linking township and village facilities with higher-level hospitals (2, 5, 6). County-level hospitals therefore serve as a critical connection within regional care delivery, and their service and management capacity is relevant to the accessibility, continuity, quality, and efficiency of local care (5–8).

High-level medical professionals are important to clinical decision making, specialty development, teaching, and hospital management in county-level settings. In this study, they were operationally defined as formally employed staff holding either a doctoral degree or a deputy senior or higher professional title. This operational definition reflects the responsibilities represented in the available workforce data; it is not a nationally standardized occupational category.

Compared with large tertiary hospitals at the provincial or municipal level, county-level hospitals may have fewer financial, research, specialty-platform, and career-development resources and may also be less attractive as workplaces (5, 8). Reviews of hospital doctors and rural physicians identify working conditions, professional development, financial and career factors, personal circumstances, and community or living conditions as interrelated influences on retention (9–13). Turnover can disrupt continuity, increase replacement costs, and add workload and stress for remaining staff (9, 10).

Evidence on turnover and retention among health professionals identifies job demands, employment conditions, organizational culture and support, work relationships, career development, job satisfaction, work-life balance, and personal circumstances as relevant factors (9–13). Evidence from China includes a systematic review and meta-analysis of primary health workers (14), studies of public-hospital physicians in central China and Guangdong (15, 16), a person-environment fit study of medical professionals in Shanghai (17), and multilevel studies of organizational culture and incentives among primary-care providers and family doctors (18, 19). A two-wave study conducted in 12 tertiary public hospitals in Guangzhou examined the moderating role of career shocks in the relationships among job embeddedness, job satisfaction, and turnover intention (20). A meta-analysis found a moderate negative association between nurses' job embeddedness and turnover intention, with on-the-job embeddedness showing a stronger association than off-the-job embeddedness (21). Most of this evidence estimates net associations. Configurational evidence is more limited: Wang et al. examined absent job-embeddedness dimensions among nurses (22), and Li et al. analyzed combinations of job demands, job resources, and personal resources among primary public health workers in Liaoning Province (23).

County-level hospitals differ in fiscal capacity, workforce composition, specialty platforms, management processes, and regional resources. These contextual differences make it useful to examine combinations of perceived conditions. At the same time, substantial correlation among conditions can make apparently distinct configurations overlap; therefore, empirical separability and unique coverage must be assessed before interpreting configurations as resource substitution.

Job Embeddedness Theory provides an appropriate framework for understanding this complexity. Mitchell et al. proposed that employees' decisions to remain in an organization depend not only on job satisfaction and organizational commitment but also on the extent to which they are embedded in their organization and community (24). Job embeddedness consists of three central dimensions: fit, links, and sacrifice, and has been developed as a theoretical foundation for comprehensive nurse retention planning (25). Meta-analytic and review evidence indicates that job embeddedness predicts turnover outcomes beyond traditional attitudinal variables and that both organizational and community contexts matter (26, 27). Health-workforce studies have also applied the construct to nurse retention and retention planning (28, 29). Fit refers to the compatibility between an individual's values, abilities, career goals, and organizational environment; links refer to formal and informal connections with colleagues, supervisors, patients, institutions, and communities; and sacrifice refers to the perceived material, professional, relational, and psychological costs of leaving the organization (24–29).

Job Embeddedness Theory organizes the study conditions into fit, links, and sacrifice. Personal development perception represents fit with professional aspirations and advancement opportunities. Institutional and environmental support and organizational and interpersonal empowerment represent formal and interpersonal links. Compensation and incentive support and internal and external platform support represent resources and opportunities that may be sacrificed on leaving. The mapping is conceptual because all conditions were measured as individual perceptions.

The study therefore treats the five dimensions as related domains rather than independent resources. Their combinations may be associated with lower turnover intention, but cross-sectional configurations cannot establish causal substitution or actual retention behavior.

fsQCA evaluates set-theoretic necessity and sufficiency, conjunctural patterns, equifinality, and causal asymmetry (30–34). In this study, the individual respondent was the case. Core conditions were those shared by the complex and parsimonious solutions; conditions appearing only in the complex solution were classified as peripheral.

Accordingly, this study combined literature review, three Delphi rounds, split-sample EFA and CFA, and fsQCA. It asked whether the five perceived domains were empirically distinguishable, whether any single condition was necessary for a high or non-high retention-oriented score, and which combinations were associated with the outcome.

The present study addresses a distinct population and setting by focusing on high-level multidisciplinary professionals in county-level hospitals. It develops county-context items through Delphi consultation, compares correlated, single-factor, second-order, and bifactor measurement models, and examines large-sample threshold and calibration sensitivity. The design therefore integrates context-specific instrument development, independent split-sample psychometric evaluation, and configurational analysis without implying causal superiority over other analytical approaches.

## 2 Materials and methods

### 2.1 Study design and settings

This multicenter cross-sectional study was conducted from July 2024 to February 2025 in 10 county-level public hospitals in Jiangsu Province. Five hospitals were in Nanjing: two in Lishui District and one each in Jiangning, Gaochun, and Liuhe districts. The other five were outside Nanjing: one each in Changzhou, Wuxi, and Suzhou and two in Yangzhou, including one in Yizheng. Hospitals were selected purposively and by convenience based on feasibility and collaboration. Hospital names were anonymized, and hospital identifiers were not retained in the final analytical dataset; consequently, hospital-specific distributions, intraclass correlation coefficients (ICCs), design effects, and hospital-level analyses could not be estimated.

### 2.2 Participants and definition of high-level medical professionals

In accordance with China's professional title system, the workforce structure of county-level hospitals, and county-level service needs, high-level medical professionals were operationally defined as formally employed staff members who held either a doctoral degree or a deputy senior or higher professional title and who undertook key professional, technical, academic, or managerial responsibilities in clinical care, nursing, pharmacy, medical technology, research and teaching, discipline development, or hospital management. Participants were required to take part voluntarily and complete a valid questionnaire. Short-term trainees, interns, standardized residency trainees, and employees on long-term leave during the survey period were excluded.

After eligibility screening, all 579 respondents included in the analysis met this operational definition.

### 2.3 Framework development and Delphi consultation

**Figure 1:**
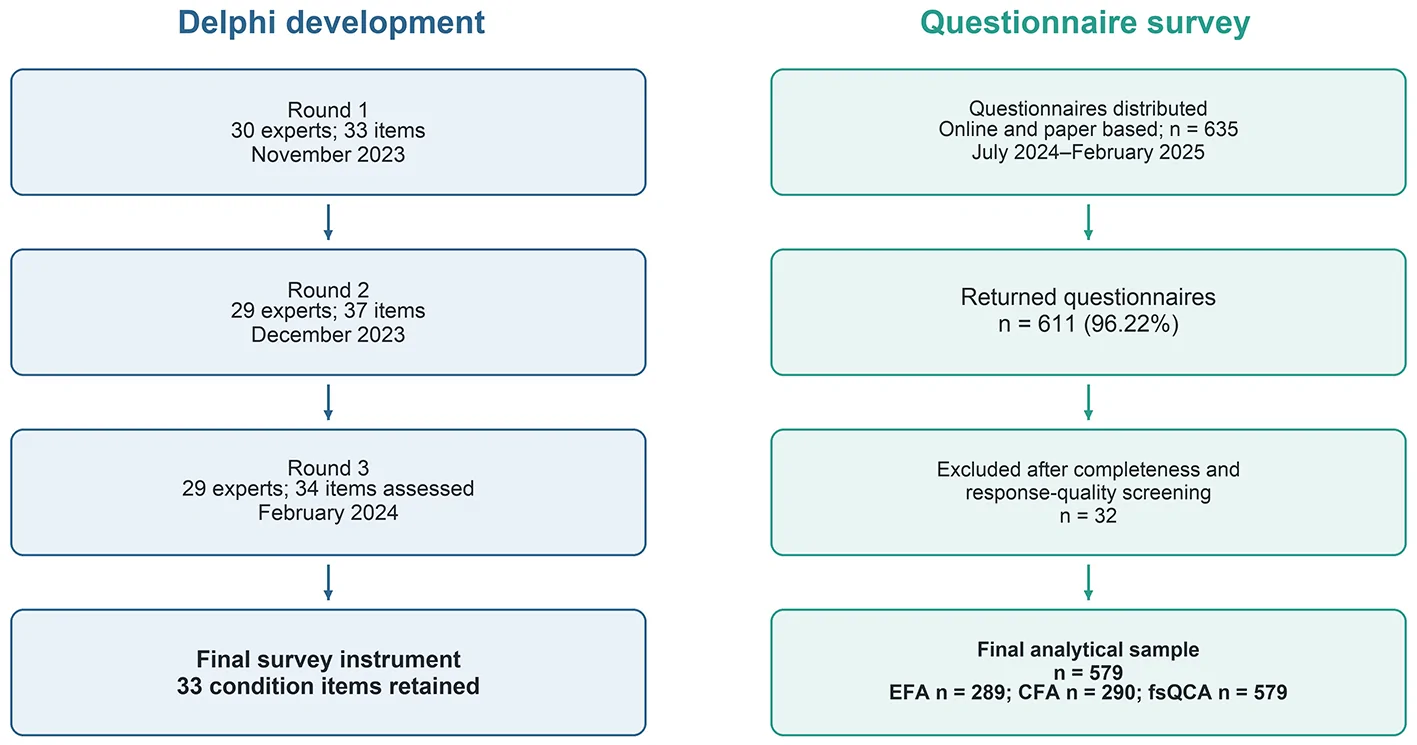
Development and survey flow.

The initial item pool was derived from literature on medical workforce retention, turnover intention, and hospital human resource management, then adapted to county-level practice. The Delphi process was designed and reported with reference to established methodological and reporting guidance (35–39). Three Delphi rounds were conducted in November 2023, December 2023, and February 2024. Thirty experts participated in round 1; one expert retired before the subsequent rounds, leaving 29 experts in rounds 2 and 3 (panel retention 96.7%). Experts were required to have at least 10 years of relevant experience in clinical care, nursing, pharmacy, medical technology, or hospital management and to be familiar with county-level healthcare delivery, workforce management, or discipline development. Twenty-five experts held deputy senior or senior professional titles. Five experts with intermediate titles were purposively included because each had at least 10 years of frontline professional or managerial experience and could provide practice-based perspectives complementary to those of senior experts. The process comprised 33 items in round 1, 37 in round 2, 34 assessed in round 3, and 33 retained for the survey (Figure 1).

Expert engagement was summarized by response rate. Authority was assessed as Cr = (Ca + Cs)/2, where Ca is the judgment-basis coefficient and Cs the familiarity coefficient. The scoring rules are shown in Table 1, and the expert-group coefficients are reported in Table 2. Consensus was assessed using the coefficient of variation and Kendall's W. The displayed overall Ca, Cs, and Cr values were calculated as arithmetic means of the primary-indicator values in Table 2.

**Table 1:** Quantitative scoring of expert judgment basis and familiarity.

Judgment basis	Influence on expert judgment			Familiarity score	
	High	Moderate	Low		
Work experience	0.5	0.4	0.3	Very familiar	1.0
Theoretical analysis	0.3	0.2	0.1	Relatively familiar	0.8
Knowledge of domestic and international peers	0.2	0.15	0.1	Moderately familiar	0.6
Intuition	0.05	0.05	0.05	Slightly familiar	0.4
				Not familiar at all	0.2

**Table 2:** Expert judgment-basis, familiarity, and authority coefficients.

Primary indicator	Judgment basis	Familiarity	Authority coefficient
Personal perception	0.81	0.93	0.87
Work environment	0.82	0.92	0.87
Management system	0.83	0.90	0.87
Compensation and incentives	0.84	0.87	0.86
Career development	0.83	0.90	0.87
Leadership style	0.83	0.86	0.85
Interpersonal relationships	0.83	0.89	0.86
Hospital platform	0.83	0.88	0.86
External factors	0.80	0.82	0.81
Mean	0.82	0.89	0.86

Overall values in Table 2 are arithmetic means of the nine displayed primary-indicator values; Ca = 0.82, Cs = 0.89, and Cr = 0.86.

Experts rated item importance on a 5-point scale. After each round, the research team summarized the mean score, coefficient of variation, full-score ratio, Kendall's W, and anonymous expert comments. The aggregate results and proposed item revisions were incorporated into the next-round questionnaire for reassessment. Items with a mean <3.5, coefficient of variation >0.25, or full-score ratio <0.50 were reviewed together with the written comments. Following round 1, items concerning team-member complementarity, cross-department coordination, and the service efficiency of functional-department leaders were added; the external-opportunity item was revised to assess differences in compensation, benefits, and development opportunities relative to peer hospitals. Items concerning an internal administrative post, family harmony, hospital grade, and the influence of the superior hospital in a medical consortium were subsequently removed. A perceived compensation-fairness item added after round 2 was removed after round 3. The consultation ended after the planned third round, when the retained items met the prespecified screening criteria and no further substantive revision was required.

### 2.4 Questionnaire survey and quality control

Data were collected using online and paper-based questionnaires. Local recruitment and questionnaire distribution were coordinated by the participating hospitals through their human resources departments or designated contacts. These contacts screened potentially eligible staff using the same prespecified inclusion and exclusion criteria and distributed 635 questionnaires. The information page described the study purpose, voluntary participation, anonymity, confidentiality, and the right to withdraw. Participants received no financial or non-financial incentives. Voluntary completion and submission of the online questionnaire or completion and return of the paper questionnaire constituted informed consent. No names, employee numbers, telephone numbers, or other direct identifiers were collected. The analytical file did not retain survey mode, hospital, department, or an identifier that could be used to verify duplicate submissions.

A total of 635 questionnaires were distributed and 611 were returned (response rate 96.22%). Thirty-two returned questionnaires that did not meet the prespecified completeness and response-quality criteria were excluded, producing 579 valid questionnaires (94.76% of returned questionnaires; 91.18% of all distributed questionnaires). The final dataset contained no missing item responses. A *post-hoc* audit identified 62 respondents with zero variance across the 31 retained condition items; 45 of these also had zero variance across the four stored, retention-oriented outcome variables. However, Y1, Y2, and Y4 had already been reverse-transformed in the stored file, so the 45 cases were not same-option responders across the mixed-worded administered outcome items. They were therefore retained in the primary analysis. Sensitivity analyses excluded the 45 stored-score-invariant cases (*n* = 534) and all 62 condition-invariant cases (*n* = 517).

The archived screening record preserved only the aggregate count of 32 exclusions and did not preserve reliable, mutually exclusive counts for individual exclusion reasons; reason-specific counts were therefore not reconstructed retrospectively. For descriptive hospital-tenure summaries, the free-text entries ‘4半', ‘1个月' (two records), and ‘37整' were converted to 4.5 years, 1/12 year, and 37 years, respectively. Two arithmetically impossible tenure values (age 28 with 18 years and age 51 with 49 years) were set to missing for the tenure summary only; all other responses were retained. Eligibility was determined from qualifications and professional titles at the time of the survey, not at first employment, so an implied employment-start age below 22 was not itself treated as invalid.

### 2.5 Outcome variable and direction of measurement

Lower turnover intention was assessed using a contextually adapted Chinese version of the four-item intention-to-quit measure reported by Farh et al. (40). Because existing Chinese wording was used, no new formal forward–backward translation was undertaken. For the present report, the Chinese wording was checked against the English wording reported by Farh et al., and the organizational referents were reviewed by the research team for clarity in the hospital context. Farh et al. used a 7-point response format; in the present questionnaire, each item was rated on a 5-point Likert scale from 1 (strongly disagree) to 5 (strongly agree), and organizational referents were adapted to the current hospital/organization context. The administered items assessed frequent thoughts of quitting (Y1), the possibility of leaving for another job in the following year (Y2), plans for long-term career development in the organization (Y3), and unfavorable future prospects if one remained (Y4). During preprocessing, the original responses to the negatively worded Y1, Y2, and Y4 were transformed as 6 minus the administered response; Y3 was retained in its original positive polarity. The deposited analytical file therefore stores Y1_R_stored, Y2_R_stored, Y3_stored, and Y4_R_stored in the same retention-oriented direction. No additional reversal was applied during analysis; the four stored variables were averaged, with higher values indicating lower turnover intention. The complete Chinese-English item bank distinguishes administered wording from stored polarity (Supplementary Table S2). This derived measure is termed a retention-oriented score and is not interpreted as observed retention or as a conceptually distinct retention-intention instrument. Cronbach's alpha was 0.917.

### 2.6 Statistical analysis

Analyses used all 579 valid cases unless otherwise stated. The 31 retained condition items yielded approximately 18.7 observations per item. Using random seed 20260809, respondents were randomly allocated to EFA (*n* = 289) and CFA (*n* = 290) subsets. EFA used minimum residual common-factor extraction with oblimin rotation. Factor retention was evaluated by 500-replication parallel analysis, requiring an observed eigenvalue to exceed the random 95th percentile (41, 42). Scale-development guidance and methods for ordered categorical data further informed item evaluation and factor retention (43, 44). An item was flagged when its primary loading was <0.40, a secondary loading was > = 0.30, or the absolute loading gap was <0.20. Because theory and the full-sample sensitivity analysis suggested five domains, a theory-guided five-factor solution was also examined transparently after reporting the parallel-analysis result.

CFA was performed in Python using semopy 2.3.11 on the independent *n* = 290 subset (45). Five-point indicators were estimated using robust maximum likelihood (MLW), Satorra-Bentler scaled chi-square, and sandwich robust standard errors; evidence comparing estimators for ordinal indicators was considered when interpreting this choice and its limitations (46). Missing-data handling was not required because the final dataset was complete. Correlated five-factor, single-factor, second-order, and orthogonal bifactor/unmeasured common-latent-factor models were compared using chi-square, df, chi-square/df, CFI, TLI, RMSEA with 90% CI, SRMR, and NFI. Reliability, composite reliability, average variance extracted, inter-factor correlations, and heterotrait-monotrait (HTMT) ratios were examined using established reliability and validity guidance (47–49). Bifactor indices were interpreted using published psychometric recommendations rather than overall fit alone (50). The single-factor and bifactor comparisons were treated as statistical diagnostics of common method variance; they cannot eliminate common method bias without a marker variable or multi-source data (51).

fsQCA used direct logistic calibration and respondent-level cases. Primary anchors were the empirical 95th, 50th, and 5th percentiles calculated using the lower-percentile definition; exact crossover memberships were set to 0.501. The frequency threshold was selected as the smallest integer that retained approximately 75%−80% of cases: 10 under the primary percentile calibration (79.1%) and 23 after re-deriving the threshold under each substantive calibration (77.9%). Because all primary 95th percentiles equaled 5, substantive-anchor sensitivity analyses used 4/3/2 and 4.5/3/1.5. Necessity required consistency > = 0.90 and was accompanied by coverage and Relevance of Necessity (RoN). For each crisp truth-table row, consistency and PRI were calculated from fuzzy minterm membership among cases assigned to that row. The primary truth table used frequency > = 10, consistency > = 0.85, and PRI > = 0.70. The complex solution is reported; comparison with the parsimonious solution identified core and peripheral conditions. Directional expectations favored the presence of each support condition for high scores and its absence for non-high scores. Robustness analyses varied frequency, consistency, PRI, calibration, crossover handling, and response-quality exclusions following established QCA guidance (30–34, 52).

## 3 Results

### 3.1 Results of the Delphi consultation

Round 1 included 30 experts (16 men and 14 women); rounds 2 and 3 included 29 after one expert retired. The first-round profile comprised 18 clinical professionals and 12 administrative managers; 10 held bachelor‘s, 16 master's, and four doctoral degrees. Five experts held intermediate titles, 16 deputy senior titles, and nine senior titles. All five intermediate-title experts had at least 10 years of relevant frontline professional or managerial experience and were included to complement the senior experts with practice-based perspectives. The item sequence was 33 -> 37 -> 34 assessed -> 33 retained. Table 3 reports item counts and Kendall's W by round, and Table 4 summarizes the characteristics of the 579 survey respondents.

**Table 3:** Kendall's coefficients of concordance and item counts across the three Delphi rounds.

Round	Assessment level	Items assessed	Kendall's W	Chi-square	df	*P* value
Round 1	Primary-indicator	9	0.316	75.899	8	<0.001
Round 1	Secondary-item	33	0.226	216.701	32	<0.001
Round 2	Primary-indicator	9	0.320	74.300	8	<0.001
Round 2	Secondary-item	37	0.388	405.412	36	<0.001
Round 3	Primary-indicator	9	0.329	76.398	8	<0.001
Round 3	Secondary-item	34	0.403	385.25	33	<0.001

Round 3 evaluated 34 secondary items (df = 33); one item was deleted after the round, leaving 33 items in the survey questionnaire.

**Table 4:** Participant characteristics (*N* = 579).

Variable	Category/statistic	*n*	Value
Age, years	Mean (SD); median [IQR]; range	579	44.49 (5.92); 44 [40-48]; 28-61
Years in hospital	Mean (SD); median [IQR]; range	577	19.24 (8.21); 18 [15-25]; 0.08-38
Sex	Female	309	53.37%
Sex	Male	270	46.63%
Ethnicity	Han	572	98.79%
Ethnicity	Ethnic minority	7	1.21%
Education	Bachelor's	382	65.98%
Education	Master's	165	28.50%
Education	Doctorate	32	5.53%
Professional title	Junior	1	0.17%
Professional title	Intermediate	3	0.52%
Professional title	Deputy senior	424	73.23%
Professional title	Senior	151	26.08%
Employment	Established post	515	88.95%
Employment	Non-established post	64	11.05%

Hospital and department distributions cannot be reported because these identifiers were not retained in the analytical dataset. All four respondents below deputy-senior title held doctoral degrees and therefore met the operational eligibility definition. The tenure summary excludes the two arithmetically impossible tenure values described in the Methods (*n* = 577).

Across the nine primary indicators, the displayed arithmetic means were Ca = 0.82, Cs = 0.89, and Cr = 0.86 (Table 2). These values indicate adequate expert authority; all cells are reported to two decimals.

At the primary-indicator level, Kendall's W changed little across rounds (0.316, 0.320, and 0.329). At the secondary-item level, W increased from 0.226 to 0.388 and 0.403. The final value indicates fair-to-moderate, not strong, concordance. The round-3 chi-square of 385.25 with df = 33 corresponds to 34 assessed secondary items; one item was subsequently removed, leaving 33 survey items.

### 3.2 Exploratory dimension extraction

The final sample was randomly divided into EFA (*n* = 289) and CFA (*n* = 290) subsets. In the EFA subset, KMO was 0.956 and Bartlett's test was significant (chi-square = 8660.44, df = 465, *P* < 0.001). Parallel analysis suggested two factors. However, the theory-guided five-factor minres-oblimin solution yielded interpretable clusters matching the five domains for most items (Table 5), with correlated factors (absolute correlations approximately 0.51–0.68). The five factors accounted for 55.4% of the item variance in the split sample. In sensitivity analyses, parallel analysis suggested five factors in the full *n* = 579 sample and in the *n* = 534 response-quality subset. The inconsistency across samples indicates that the five-domain structure is preliminary rather than definitive.

**Table 5:** Pattern matrix for the theory-constrained five-factor EFA solution (*n* = 289).

Secondary item	PD	IES	CIS	OIE	IEPS
Fit between the position and county-level service needs	0.746	0.019	0.109	−0.066	−0.035
Sense of achievement in primary clinical service	0.762	−0.013	0.063	−0.067	0.106
Professional recognition of county-level health professionals	0.737	−0.026	0.012	0.006	0.107
Internal promotion pathways in county-level hospitals	0.603	0.205	−0.007	0.179	−0.029
Practical skills training for primary care	0.574	0.115	0.012	0.267	–.032
Support for continuing education of county-level staff	0.590	0.207	−0.025	0.212	−0.001
Adequacy of facilities for primary clinical care	0.083	0.620	0.022	−0.023	0.083
Timeliness of logistical support in county-level hospitals	0.030	0.765	0.067	−0.041	−0.003
Accessible channels for frontline staff participation in hospital management	0.077	0.698	−0.017	0.071	−0.007
Complementarity of professional skills within county-level teams	0.110	0.640	−0.004	0.028	0.053
Adaptation and streamlining of clinical and administrative processes	0.031	0.811	0.028	0.014	0.044
Fair implementation of institutional policies for county-level staff	−0.003	0.740	0.070	0.028	0.067
Validity of performance indicators adapted to county-level work	−0.026	0.852	0.041	−0.017	0.047
Effectiveness of interdepartmental collaboration in county-level hospitals	0.041	0.772	0.013	0.074	0.033
Adequacy of base compensation for county-level medical positions	0.046	−0.130	0.898	0.010	−0.008
Alignment between primary-care workload and compensation	0.080	0.062	0.805	−0.049	0.071
Provision of paid leave and rotational rest for county-level staff	−0.063	0.087	0.762	0.081	−0.046
Targeted incentives for county-level professionals	−0.025	0.139	0.781	0.061	0.027
Hospital leaders' understanding of and decision-making capacity for county-level health needs	−0.035	0.182	0.099	0.567	0.116
Department heads' professional guidance and humanistic support	0.044	−0.030	0.028	0.730	0.163
Efficiency of administrative departments in supporting frontline clinical services	0.018	0.311	0.029	0.519	0.066
Quality of clinician–patient communication in county-level settings	0.042	0.154	0.094	0.594	−0.018
Positive working relationships among colleagues	0.073	0.065	0.042	0.727	−0.016
Collaborative trust between supervisors and staff in clinical tasks	0.050	−0.047	0.093	0.779	0.056
Recognition of medical professions by family members and county residents	0.167	−0.128	0.098	0.465	0.282
Academic exchange and research support in county-level hospitals	−0.065	0.184	0.042	0.078	0.595
Development of distinctive specialties in county-level hospitals	−0.070	0.024	0.040	0.148	0.761
Local workforce retention and development mechanisms in county-level hospitals	0.005	0.106	−0.035	0.110	0.672
County economic development and health investment	−0.010	0.055	0.004	0.014	0.879
Hospital competitiveness within the county and surrounding areas	0.095	−0.067	−0.007	−0.027	0.899
Gap in compensation, benefits, and development opportunities compared with peer hospitals	0.050	0.052	0.066	−0.060	0.808
SS loadings	2.826	4.774	2.723	3.055	3.800
Variance explained (%)	9.1	15.4	8.8	9.9	12.3
First five observed eigenvalues (descending)	17.207	1.817	1.504	1.486	1.128

Minimum residual common-factor extraction with oblimin rotation. Parallel analysis suggested two factors in the split EFA sample; the displayed five-factor solution was retained for theory-guided comparison. The five observed eigenvalues are listed in descending order and are not assigned to the rotated factor columns. KMO = 0.956; Bartlett's chi-square = 8660.44, df = 465, *P* < 0.001.

The five retained domains were personal development perception (PD; six items), institutional and environmental support (IES; eight items), compensation and incentive support (CIS; four items), organizational and interpersonal empowerment (OIE; seven items), and internal and external platform support (IEPS; six items). Two items concerning workload/administrative burden and internal collaboration climate were excluded before the 31-item condition score was finalized. The empirical five-domain grouping reorganized the nine Delphi primary indicators; this reduction suggests that experts differentiated content more finely than respondents did statistically.

**Figure 2:**
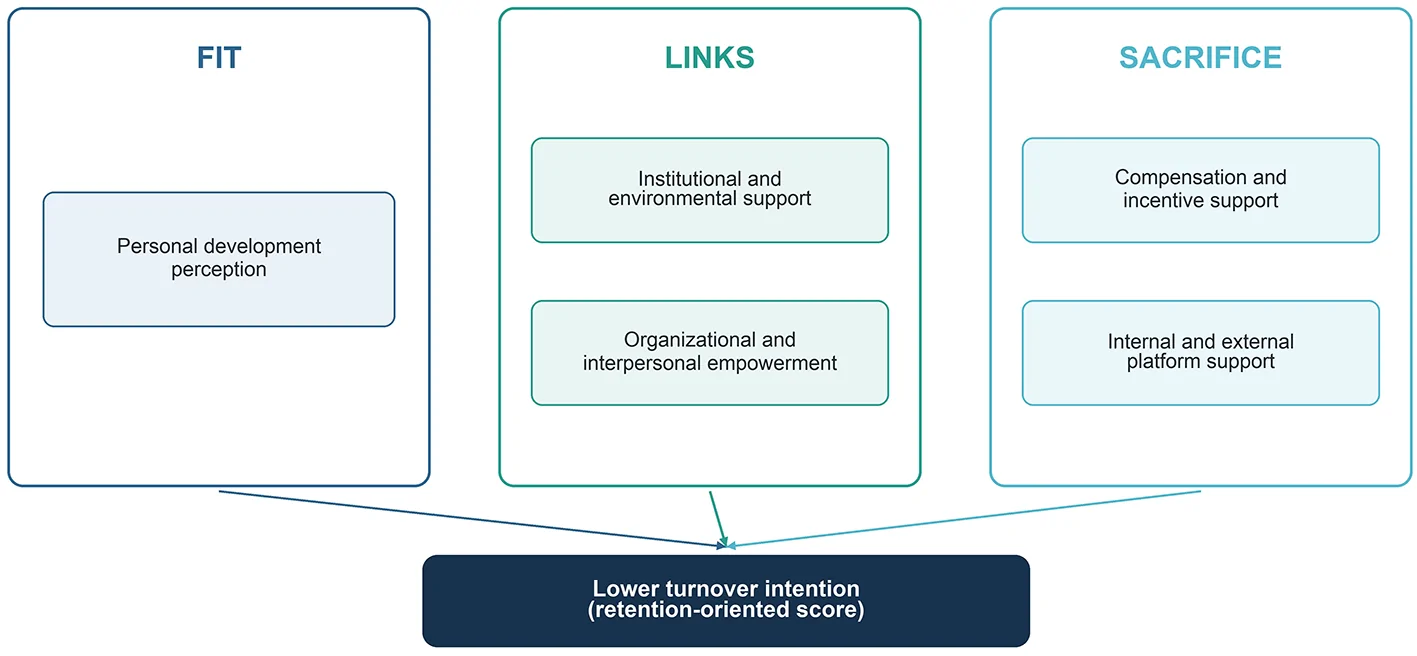
Conceptual mapping of the five perceived condition domains to fit, links, and sacrifice.

Accordingly, the five domains were retained as theory-guided, correlated condition sets for fsQCA, not interpreted as fully independent constructs. Figure 2 shows their conceptual mapping to fit, links, and sacrifice.

### 3.3 Confirmatory factor analysis and measurement quality

Internal consistency was high for PD (alpha = 0.921), IES (0.950), CIS (0.916), OIE (0.945), IEPS (0.940), and the four-item outcome (0.917). Alpha across all 31 condition items was 0.971. This value supports internal consistency but also indicates possible item redundancy or a strong general factor.

Robust CFA results are shown in Table 6. The correlated five-factor model had marginal fit: Satorra-Bentler chi-square = 1350.48, df = 424, chi-square/df = 3.19, CFI = 0.903, TLI = 0.893, RMSEA = 0.087 (90% CI 0.082-0.092), and SRMR = 0.042. It fit substantially better than the single-factor model but was nearly indistinguishable from the second-order model. The bifactor model improved CFI only slightly to 0.907 and did not materially reduce RMSEA or SRMR.

**Table 6:** Comparison of robust CFA models in the validation sample (*n* = 290).

Model	SB chi-square	df	chi-square/df	CFI	TLI	RMSEA (90% CI)	SRMR
Five-factor correlated	1350.48	424	3.19	0.903	0.893	0.087 (0.082–0.092)	0.042
Single-factor	4037.42	434	9.30	0.621	0.594	0.169 (0.165–0.174)	0.102
Second-order	1372.79	429	3.20	0.901	0.893	0.087 (0.082–0.092)	0.046
Bifactor	1289.29	403	3.20	0.907	0.893	0.087 (0.082–0.093)	0.042

Standardized five-factor loadings, AVE, and CR are reported in Table 7. The bifactor diagnostics showed explained common variance = 0.657, omega hierarchical = 0.889, omega total = 0.984, and percentage of uncontaminated correlations = 0.817. These results indicate a strong general factor alongside domain-specific variance. Therefore, the five domains are useful for content organization, but claims of empirical independence or broad resource substitution are not warranted.

**Table 7:** Robust standardized loadings, average variance extracted, and composite reliability (CFA *n* = 290).

Latent factor	Secondary item	Std. loading	Robust S.E.	*P*	AVE	CR
Personal development perception	Fit between the position and county-level service needs	0.706	–	–	0.675	0.925
	Sense of achievement in primary clinical service	0.784	0.065	<0.001		
	Professional recognition of county-level health professionals	0.781	0.068	<0.001		
	Internal promotion pathways in county-level hospitals	0.876	0.091	<0.001		
	Practical skills training for primary care	0.856	0.097	<0.001		
	Support for continuing education of county-level staff	0.909	0.094	<0.001		
Institutional and environmental support	Adequacy of facilities for primary clinical care	0.770	–	–	0.738	0.957
	Timeliness of logistical support in county-level hospitals	0.854	0.061	<0.001		
	Accessible channels for frontline staff participation in hospital management	0.802	0.082	<0.001		
	Complementarity of professional skills within county-level teams	0.841	0.064	<0.001		
	Adaptation and streamlining of clinical and administrative processes	0.893	0.060	<0.001		
	Fair implementation of institutional policies for county-level staff	0.902	0.064	<0.001		
	Validity of performance indicators adapted to county-level work	0.907	0.076	<0.001		
	Effectiveness of interdepartmental collaboration in county-level hospitals	0.896	0.089	<0.001		
Compensation and incentive support	Adequacy of base compensation for county-level medical positions	0.812	–	–	0.732	0.916
	Alignment between primary-care workload and compensation	0.936	0.078	<0.001		
	Provision of paid leave and rotational rest for county-level staff	0.747	0.084	<0.001		
	Targeted incentives for county-level professionals	0.913	0.086	<0.001		
Organizational and interpersonal empowerment	Hospital leaders' understanding of and decision-making capacity for county-level health needs	0.883	–	–	0.746	0.953
	Department heads' professional guidance and humanistic support	0.901	0.039	<0.001		
	Efficiency of administrative departments in supporting frontline clinical services	0.893	0.028	<0.001		
	Quality of clinician–patient communication in county-level settings	0.803	0.045	<0.001		
	Positive working relationships among colleagues	0.878	0.045	<0.001		
	Collaborative trust between supervisors and staff in clinical tasks	0.885	0.042	<0.001		
	Recognition of medical professions by family members and county residents	0.797	0.054	<0.001		
Internal and external platform support	Academic exchange and research support in county-level hospitals	0.779	–	–	0.729	0.942
	Development of distinctive specialties in county-level hospitals	0.884	0.056	<0.001		
	Local workforce retention and development mechanisms in county-level hospitals	0.847	0.062	<0.001		
	County economic development and health investment	0.907	0.056	<0.001		
	Hospital competitiveness within the county and surrounding areas	0.858	0.067	<0.001		
	Gap in compensation, benefits, and development opportunities compared with peer hospitals	0.843	0.073	<0.001		

Robust standard errors use sandwich estimation. Reference indicators have their unstandardized loading fixed at 1.

AVE, average variance extracted; CR, composite reliability.

Composite correlations ranged from 0.576 to 0.739, and all HTMT ratios were below 0.85 (maximum 0.781 for IES and IEPS; Table 8). The single-factor model fit poorly, arguing against a single dimension as a complete account, but common method variance remains plausible because all variables were self-reported.

**Table 8:** Composite correlations and HTMT ratios.

Dimension	PD	IES	CIS	OIE	IEPS
PD	Alpha = 0.921	0.697	0.657	0.669	0.661
IES	0.655	Alpha = 0.950	0.677	0.734	0.781
CIS	0.606	0.633	Alpha = 0.916	0.639	0.618
OIE	0.626	0.698	0.596	Alpha = 0.945	0.717
IEPS	0.619	0.739	0.576	0.678	Alpha = 0.940

Cronbach's alpha appears on the diagonal; Pearson correlations are below the diagonal; HTMT ratios are above the diagonal.

PD, personal development perception; IES, institutional and environmental support; CIS, compensation and incentive support; OIE, organizational and interpersonal empowerment; IEPS, internal and external platform support.

### 3.4 fsQCA calibration and necessity analysis

Composite distributions and calibration anchors are reported in Tables 9, 10. Ceiling percentages ranged from 9.8 to 17.6%, with the outcome showing the largest ceiling (17.6%). Anchors were calculated using the lower-percentile definition: full-membership anchors were 5 for every set, and the crossover and full non-membership anchors followed the scored item grids shown in Table 9. The corrected high-score truth table retained 79.1% of cases at frequency > = 10 (Supplementary Table S1).

**Table 9:** Calibration anchors for conditions and the retention-oriented outcome (lower-percentile definition).

Condition/outcome	Full membership	Crossover	Full non-membership
Personal development perception	5.000	4.000	2.667
Institutional and environmental support	5.000	3.875	2.625
Compensation and incentive support	5.000	4.000	2.000
Organizational and interpersonal empowerment	5.000	4.000	3.000
Internal and external platform support	5.000	3.833	2.000
Retention-oriented outcome	5.000	4.000	2.500
Calibration (lower-percentile definition)	Direct logistic	Exact 0.5 -> 0.501	Sensitivity: 4/3/2 and 4.5/3/1.5

**Table 10:** Composite descriptives, floor and ceiling effects, and reliability (*N* = 579).

Composite	Items	Mean	SD	Median	IQR	Skewness	Kurtosis	Floor %	Ceiling %	Alpha
PD	6	3.874	0.769	4.000	0.833	−0.879	1.821	1.4	11.4	0.921
IES	8	3.802	0.709	3.875	0.750	−0.355	0.538	0.3	9.8	0.950
CIS	4	3.762	0.887	4.000	1.000	−0.859	1.114	2.9	14.0	0.916
OIE	7	3.949	0.687	4.000	0.714	−0.603	1.617	0.7	14.5	0.945
IEPS	6	3.632	0.836	3.833	1.000	−0.441	0.149	1.0	9.8	0.940
Outcome	4	3.967	0.842	4.000	1.250	−1.006	1.356	1.0	17.6	0.917

Kurtosis is excess kurtosis. Floor and ceiling are exact composite scores of 1 and 5, respectively.

**Figure 3:**
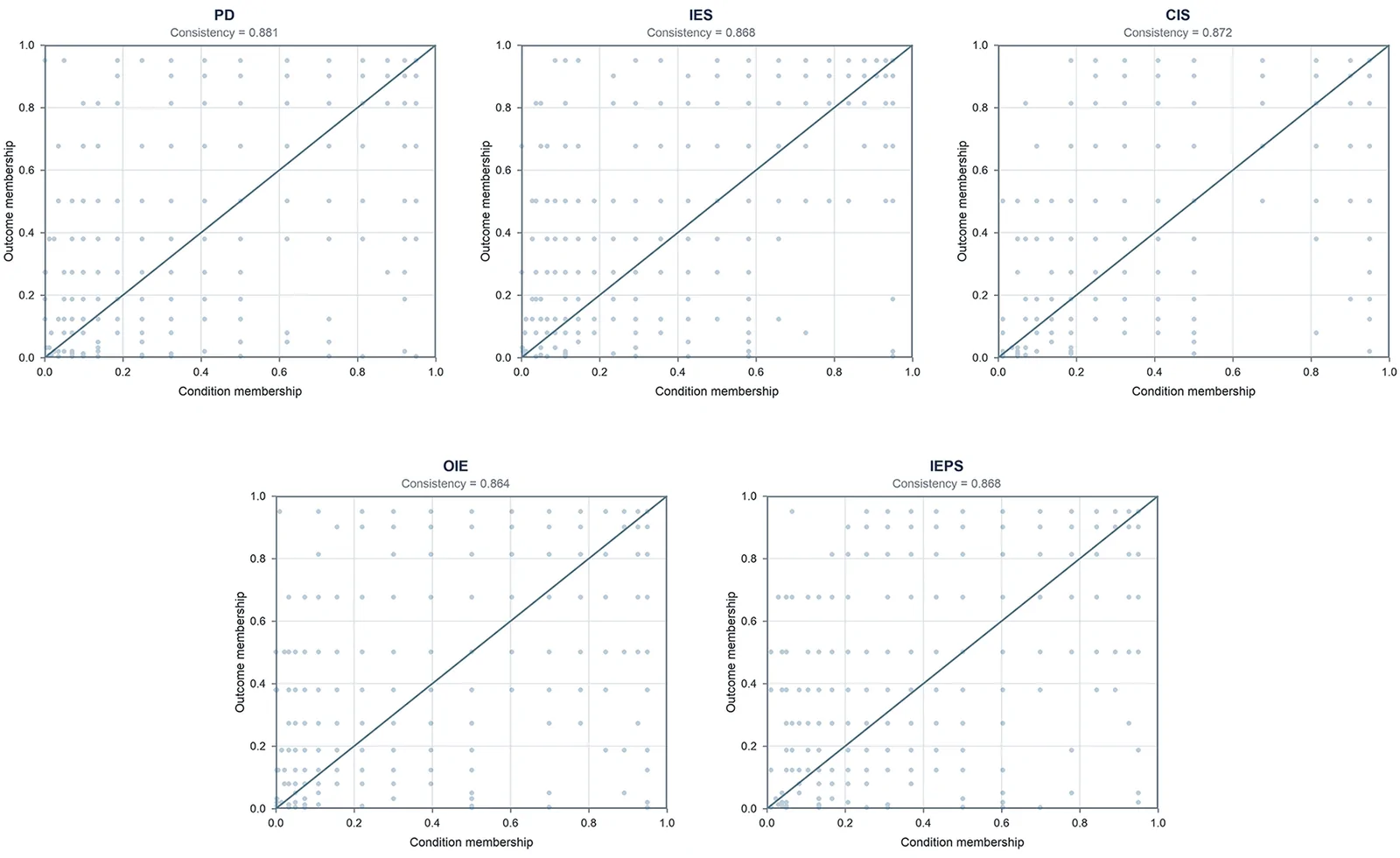
Necessity plots for the five conditions and the high retention-oriented outcome.

No condition or negation met the necessity threshold of 0.90 for either high or non-high scores (Table 11; Figure 3). For the high score, present-condition consistencies ranged from 0.864 to 0.881, with personal development perception highest at 0.881. For the non-high score, absent-condition consistencies ranged from 0.754 to 0.777. Thus, none established necessity, and the non-high values were not close to the prespecified threshold.

**Table 11:** Necessity analysis for high and non-high retention-oriented scores.

Outcome	Condition	State	Consistency	Coverage	RoN	Necessary?
High score	Personal development perception	Presence	0.881	0.787	0.899	No
High score	Personal development perception	Absence	0.570	0.544	0.685	No
High score	Institutional and environmental support	Presence	0.868	0.775	0.890	No
High score	Institutional and environmental support	Absence	0.577	0.552	0.688	No
High score	Compensation and incentive support	Presence	0.872	0.777	0.894	No
High score	Compensation and incentive support	Absence	0.590	0.565	0.694	No
High score	Organizational and interpersonal empowerment	Presence	0.864	0.769	0.888	No
High score	Organizational and interpersonal empowerment	Absence	0.581	0.556	0.689	No
High score	Internal and external platform support	Presence	0.868	0.761	0.894	No
High score	Internal and external platform support	Absence	0.589	0.572	0.687	No
Non-high score	Personal development perception	Presence	0.490	0.516	0.677	No
Non-high score	Personal development perception	Absence	0.777	0.874	0.808	No
Non-high score	Institutional and environmental support	Presence	0.498	0.524	0.681	No
Non-high score	Institutional and environmental support	Absence	0.764	0.861	0.798	No
Non-high score	Compensation and incentive support	Presence	0.512	0.537	0.688	No
Non-high score	Compensation and incentive support	Absence	0.767	0.866	0.800	No
Non-high score	Organizational and interpersonal empowerment	Presence	0.502	0.527	0.683	No
Non-high score	Organizational and interpersonal empowerment	Absence	0.759	0.857	0.794	No
Non-high score	Internal and external platform support	Presence	0.512	0.529	0.694	No
Non-high score	Internal and external platform support	Absence	0.754	0.864	0.786	No

A condition was considered necessary when consistency was at least 0.90.

RoN, relevance of necessity.

### 3.5 Configuration associated with the high score and diagnostic non-high analysis

**Figure 4:**
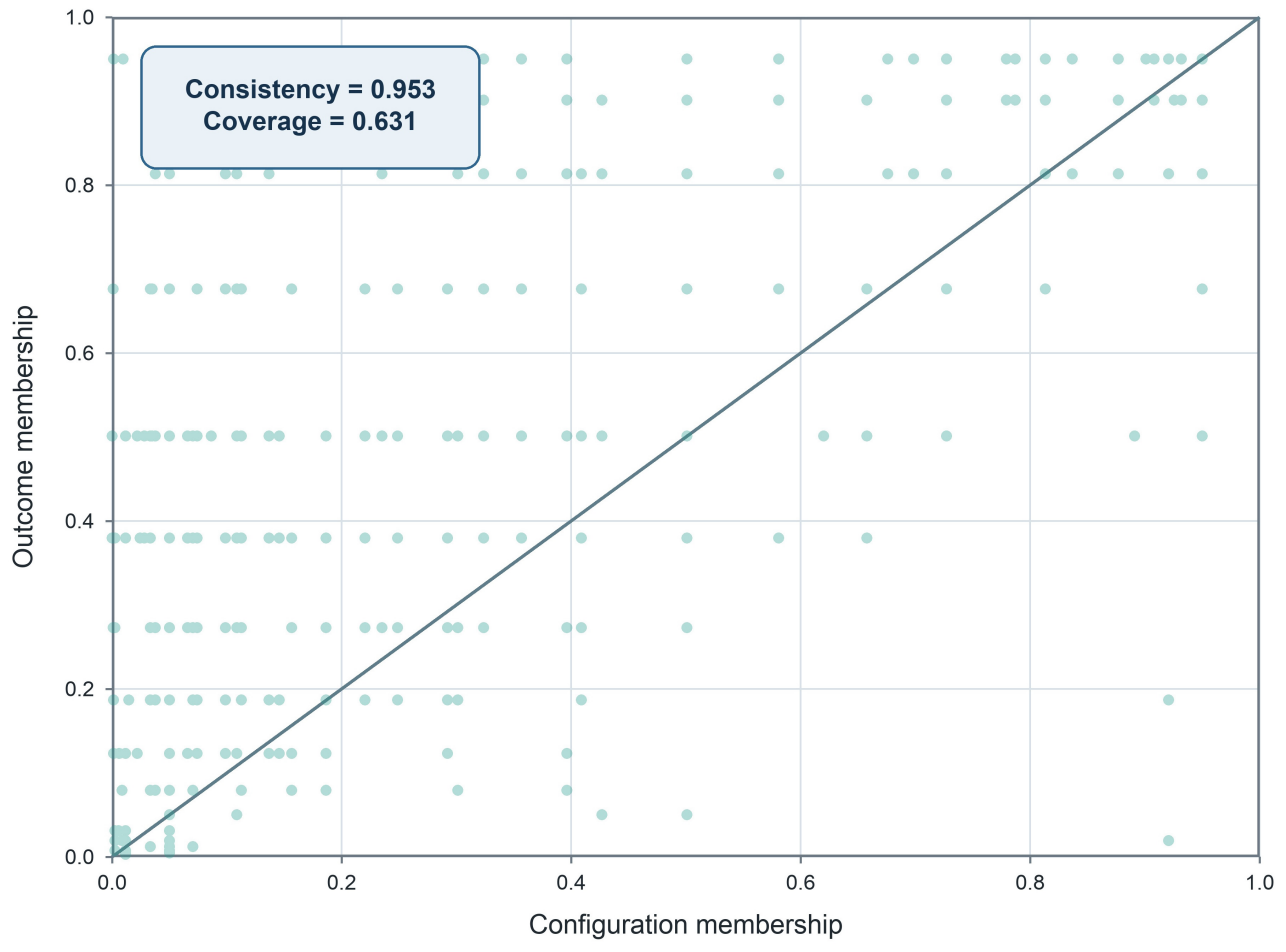
Sufficiency XY plot for the primary high-score configuration.

The corrected primary complex solution identified one configuration associated with a high retention-oriented score (Table 12; Figure 4): PD^*^IES^*^CIS^*^OIE. Its consistency was 0.953, PRI was 0.917, and raw and solution coverage were 0.631.

**Table 12:** Configuration associated with the high retention-oriented score and diagnostic non-high analysis.

Condition/metric	High-score configuration	Role	Non-high diagnostic
Personal development perception	Present	Core present	Not interpreted by condition
Institutional/environmental support	Present	Peripheral present	Not interpreted by condition
Compensation/incentive support	Present	Core present	Not interpreted by condition
Organizational/interpersonal empowerment	Present	Peripheral present	Not interpreted by condition
Internal/external platform support	Not included	Not included	Not interpreted by condition
Raw coverage	0.631	–	0.760 (solution)
Unique coverage	0.631	–	Not emphasized
Consistency	0.953	–	0.850 (solution)
PRI	0.917	–	0.695 (solution)
Expression	PD^*^IES^*^CIS^*^OIE	Single term	~PD^*^IES^*^CIS^*^OIE^*^~IEPS + ~PD^*^~IES^*^~CIS^*^~IEPS

Core and peripheral status is shown explicitly. Core conditions appear in both the complex and parsimonious solutions; peripheral conditions appear only in the complex solution. The non-high result is diagnostic only because its consistency and PRI were borderline and its expression was unstable across sensitivity settings. The symbol “*” denotes the logical AND/conjunction operator in fsQCA configuration expressions; for example, PD*IES*CIS*OIE means that these conditions are present together in the same configuration.

The parsimonious solution was PD^*^CIS. Accordingly, personal development perception and compensation/incentive support were core present conditions; institutional/environmental support and organizational/interpersonal empowerment were peripheral present conditions; internal/external platform support was not included in the primary configuration.

The single term indicates conjunction rather than substitution: all four included conditions had to be present in the minimized primary expression. Its relatively high consistency and moderate coverage describe a sufficient respondent-level pattern, not a deterministic rule or a causal intervention package.

For the non-high outcome, the corrected complex expression was ~PD^*^IES^*^CIS^*^OIE^*^~IEPS + ~PD^*^~IES^*^~CIS^*^~IEPS (solution consistency = 0.850; PRI = 0.695; coverage = 0.760). Because consistency and PRI were borderline and the expression changed across reasonable specifications, it is reported only as a diagnostic result and is not interpreted substantively.

The primary high-score configuration covered 63.1% of fuzzy outcome membership. Because the solution contained a single term, its raw, unique, and solution coverage were identical.

No stable, policy-distinct configuration was supported for non-high scores. The varying diagnostic expressions precluded a separate causal-asymmetry claim.

### 3.6 Robustness analysis

Robustness results are summarized in Table 13. The primary term PD^*^IES^*^CIS^*^OIE was unchanged when the frequency threshold was raised to 11, when consistency was varied to 0.80 or 0.90, when PRI was varied to 0.65 or 0.75, and under both substantive calibrations. At frequency 9, an additional term, PD^*^IES^*^CIS^*^~IEPS, appeared. Using the primary anchors and thresholds after excluding the 45 stored-score-invariant cases or all 62 condition-invariant cases retained the primary term. Re-deriving anchors and the frequency threshold in the n = 534 subset added PD^*^CIS^*^OIE^*^IEPS. Coding crossover ties low added PD^*^IES^*^~CIS^*^~OIE. Thus, the four-condition primary term was recurrent, but neither IES nor OIE appeared in every term across all sensitivity settings. The substantive-calibration frequency of 23 was re-derived by the same 75%−80% case-retention rule used for the primary analysis.

**Table 13:** Corrected fsQCA robustness analyses.

Specification	*n*	Frequency	Consistency	PRI	High-score expression	Solution consistency	Solution coverage	Non-high expression
Primary percentile calibration	579	10	0.85	0.70	PD^*^IES^*^CIS^*^OIE	0.953	0.631	Diagnostic; see text
Frequency threshold 9	579	9	0.85	0.70	PD^*^IES^*^CIS^*^OIE + PD^*^IES^*^CIS^*^~IEPS	0.949	0.655	Varied; diagnostic only
Frequency threshold 11	579	11	0.85	0.70	PD^*^IES^*^CIS^*^OIE	0.953	0.631	Varied; diagnostic only
Consistency 0.80	579	10	0.80	0.70	PD^*^IES^*^CIS^*^OIE	0.953	0.631	Varied; diagnostic only
Consistency 0.90	579	10	0.90	0.70	PD^*^IES^*^CIS^*^OIE	0.953	0.631	Varied; diagnostic only
PRI 0.65	579	10	0.85	0.65	PD^*^IES^*^CIS^*^OIE	0.953	0.631	Varied; diagnostic only
PRI 0.75	579	10	0.85	0.75	PD^*^IES^*^CIS^*^OIE	0.953	0.631	Varied; diagnostic only
Substantive calibration 4/3/2	579	23	0.85	0.70	PD^*^IES^*^CIS^*^OIE	0.971	0.800	No solution
Substantive calibration 4.5/3/1.5	579	23	0.85	0.70	PD^*^IES^*^CIS^*^OIE	0.974	0.806	No solution
Exclude 45; fixed primary specification	534	10	0.85	0.70	PD^*^IES^*^CIS^*^OIE	0.943	0.583	Varied; diagnostic only
Exclude 45; anchors/threshold re-derived	534	11	0.85	0.70	PD^*^IES^*^CIS^*^OIE + PD^*^CIS^*^OIE^*^IEPS	0.940	0.625	Varied; diagnostic only
Exclude all 62 condition-invariant cases	517	10	0.85	0.70	PD^*^IES^*^CIS^*^OIE	0.945	0.580	Varied; diagnostic only
Crossover ties coded low	579	10	0.85	0.70	PD^*^IES^*^CIS^*^OIE + PD^*^IES^*^~CIS^*^~OIE	0.935	0.673	Varied; diagnostic only

The primary four-condition term recurred across most specifications, but additional terms appeared at frequency 9, after fully re-deriving the *n* = 534 specification, and when crossover ties were coded low. The non-high results were substantially less stable. Frequency 23 under the substantive calibrations was re-derived using the same approximately 75%−80% case-retention rule. The symbol “*” denotes the logical AND/conjunction operator in fsQCA configuration expressions; for example, PD*IES*CIS*OIE means that these conditions are present together in the same configuration.

## 4 Discussion

### 4.1 Principal findings

This study found qualified support for five content domains but also strong evidence of a general factor. Split-sample EFA was unstable across factor-retention analyses, and five-factor CFA fit was marginal and nearly equivalent to a second-order model. The corrected fsQCA identified one high-score configuration, PD^*^IES^*^CIS^*^OIE, with PD and CIS core and IES and OIE peripheral.

No single condition was necessary. The high-score configuration indicates that favorable perceptions of personal development, institutional/environmental support, compensation/incentive support, and organizational/interpersonal empowerment occurred jointly in a sufficient respondent-level pattern. It does not demonstrate that one hospital resource causally substitutes for another.

The corrected configurational evidence therefore supports one conjunctural pattern rather than alternative pathways. The term recurred across most robustness specifications, although additional terms emerged under selected thresholds and data-handling choices. The non-high solution was not stable enough to support distinct policy claims or strong causal asymmetry.

### 4.2 Theoretical interpretation and configurational mechanisms

Within Job Embeddedness Theory, PD reflects perceived fit, IES and OIE reflect formal and interpersonal links, and CIS represents a valued resource that may be sacrificed on leaving (24–29). Their joint appearance is compatible with lower turnover intention arising from simultaneous fit, links, and sacrifice-related perceptions. IEPS was not included in the minimized primary expression, but this absence should not be interpreted as evidence that platform support is unimportant. The strong general factor also suggests that respondents may have been expressing a broad favorable or unfavorable evaluation of their workplace.

The CFA comparisons temper the configurational interpretation. The correlated five-factor model fit much better than a single factor, yet its similarity to the second-order model and the bifactor ECV of 0.657 indicate substantial common variance. The condition sets are therefore best treated as related management domains rather than empirically independent resources.

In the current data, the primary four-condition term was stable across the prespecified consistency and PRI variations, the higher frequency threshold, both substantive calibrations, and fixed-specification response-quality exclusions. Additional terms appeared at frequency 9, after fully re-deriving the *n* = 534 specification, and when crossover ties were coded low. These exact differences show why threshold selection, calibration sensitivity, and response-quality handling must be reported rather than summarized as uniform prominence of particular conditions.

The primary high-score configuration does not imply that compensation alone is sufficient. Compensation/incentive support appeared together with personal development, institutional/environmental support, and organizational/interpersonal empowerment. This conjunction is consistent with Chinese studies in which professional environment, career development, work stress, job satisfaction, organizational culture, and organizational incentives were associated with turnover intention (14–19), while remaining specific to the configurational evidence in this sample.

The non-high expressions should be read only as diagnostics. Their borderline consistency and PRI and their instability across reasonable settings do not support a distinct causal mechanism for low retention-oriented scores.

### 4.3 Public health and management implications

For county-level hospitals, the findings identify four areas for joint assessment rather than causal prescriptions: development fit, institutional processes, compensation/incentives, and organizational/interpersonal empowerment. Because the sample was cross-sectional and non-probability based, managers should examine local staff perceptions before selecting or evaluating any intervention. This diagnostic emphasis is particularly relevant because county-hospital capacity and management constraints vary across settings (5, 6, 8).

Personal-development assessment may include transparent promotion criteria, access to continuing education, practical skills training, and alignment between professional roles and county service needs. These examples correspond to PD in the configuration but were not individually tested as interventions.

Institutional and relational assessment may include procedural fairness, responsive logistical support, participation channels, leadership engagement, interdepartmental cooperation, and trust. Compensation assessment may include workload-linked pay, leave arrangements, and transparent targeted incentives. The data indicate that these domains appeared jointly; they do not show that any single measure can replace the others.

Implementation should therefore be treated as a locally adapted, multifaceted hypothesis rather than a fixed package. This cautious approach is compatible with reviews favoring multifaceted retention strategies over isolated interventions (9–13). Prospective evaluation is needed before attributing changes in turnover or retention to these measures.

Hospitals should not apply the configuration as a deterministic template. It is an overlapping respondent-level set relation from one province and is more appropriately used to structure local diagnosis and hypothesis generation than to prescribe a uniform policy package.

## 5 Limitations

This study has several limitations. Hospitals and respondents were selected non-probabilistically from 10 public county-level hospitals in Jiangsu, including five in Nanjing and five elsewhere in the province; results may not generalize to western, rural, private, or differently resourced settings in China. Hospital and department identifiers were not retained, preventing hospital distributions, measurement invariance, ICCs, design effects, cluster-adjusted estimates, or multilevel analysis. All conclusions are therefore restricted to individual perceptions. Although participation was voluntary and no incentives were provided, the high response rate and hospital-mediated distribution may still have produced perceived obligation or socially desirable responding; the mixed online and paper modes and absence of respondent identifiers prevented verification of duplicate submissions or mode-specific patterns. Sixty-two respondents were invariant across condition items, but the 45 also invariant across stored outcome scores were not same-option responders in the administered mixed-worded outcome scale because three stored variables had already been reverse-transformed. Sensitivity analyses excluding 45 and 62 cases were therefore reported. The outcome was a retention-oriented score derived from a contextually adapted, 5-point version of the Farh et al. turnover-intention measure (40), rather than actual retention or a distinct validated retention-intention construct. The cross-sectional design cannot establish temporal order or causality. Split-sample validation reduced reuse of cases, but EFA parallel analysis suggested two factors in the *n* = 289 subset and five in full-sample sensitivity analyses; CFA fit was marginal, and alpha = 0.971 plus bifactor results suggest redundancy or common variance. Single-factor and bifactor tests do not exclude common method bias (51). The primary fsQCA term was recurrent across many specifications, but additional terms and the non-high diagnostic changed under selected thresholds and preprocessing choices. Finally, target-population content validation, test-retest reliability, and prospective prediction of actual turnover remain needed.

## 6 Conclusion

Among high-level medical professionals surveyed in 10 county-level hospitals in Jiangsu, one configuration, PD^*^IES^*^CIS^*^OIE, was associated with lower turnover intention. Personal development perception and compensation/incentive support were core present conditions, and institutional/environmental support and organizational/interpersonal empowerment were peripheral present conditions; internal/external platform support was not included. The findings describe a conjunctural association but do not demonstrate causality, substitutable pathways, hospital-level effects, or actual retention. Replication with retained hospital identifiers, independent regional samples, longitudinal follow-up, and multi-source data is required.

## Data Availability

The original contributions presented in the study are included in the article/Supplementary material, further inquiries can be directed to the corresponding authors.
