# Supplementary material for: Factors and a configurational pathway associated with lower turnover intention among high-level medical professionals in county-level hospitals in China: a multicenter cross-sectional study
Source: Front Public Health. 2026 Sep 8;14:1917115. doi: 10.3389/fpubh.2026.1917115 (PMC13588790; doi:10.3389/fpubh.2026.1917115)
Supplement: Supplementary file 1 [file Table_2.docx]

**Supplementary Table S1 Corrected full truth table for the high retention-oriented outcome**

| Configuration | PD | IES | CIS | OIE | IEPS | Cases | Consistency | PRI | Retained | Outcome |
| --- | --- | --- | --- | --- | --- | --- | --- | --- | --- | --- |
| 00000 | 0 | 0 | 0 | 0 | 0 | 152 | 0.387 | 0.084 | Yes | 0 |
| 00001 | 0 | 0 | 0 | 0 | 1 | 8 | 0.752 | 0.287 | No | Logical remainder |
| 00010 | 0 | 0 | 0 | 1 | 0 | 24 | 0.572 | 0.059 | Yes | 0 |
| 00011 | 0 | 0 | 0 | 1 | 1 | 11 | 0.660 | 0.338 | Yes | 0 |
| 00100 | 0 | 0 | 1 | 0 | 0 | 9 | 0.482 | 0.001 | No | Logical remainder |
| 00101 | 0 | 0 | 1 | 0 | 1 | 1 | 1.000 | 1.000 | No | Logical remainder |
| 00110 | 0 | 0 | 1 | 1 | 0 | 7 | 0.713 | 0.427 | No | Logical remainder |
| 00111 | 0 | 0 | 1 | 1 | 1 | 4 | 0.772 | 0.469 | No | Logical remainder |
| 01000 | 0 | 1 | 0 | 0 | 0 | 10 | 0.711 | 0.476 | Yes | 0 |
| 01001 | 0 | 1 | 0 | 0 | 1 | 5 | 0.441 | 0.000 | No | Logical remainder |
| 01010 | 0 | 1 | 0 | 1 | 0 | 5 | 0.801 | 0.573 | No | Logical remainder |
| 01011 | 0 | 1 | 0 | 1 | 1 | 4 | 0.751 | 0.589 | No | Logical remainder |
| 01100 | 0 | 1 | 1 | 0 | 0 | 6 | 0.562 | 0.003 | No | Logical remainder |
| 01101 | 0 | 1 | 1 | 0 | 1 | 4 | 0.879 | 0.016 | No | Logical remainder |
| 01110 | 0 | 1 | 1 | 1 | 0 | 10 | 0.746 | 0.008 | Yes | 0 |
| 01111 | 0 | 1 | 1 | 1 | 1 | 15 | 0.827 | 0.575 | Yes | 0 |
| 10000 | 1 | 0 | 0 | 0 | 0 | 6 | 0.810 | 0.534 | No | Logical remainder |
| 10001 | 1 | 0 | 0 | 0 | 1 | 2 | 0.502 | 0.000 | No | Logical remainder |
| 10010 | 1 | 0 | 0 | 1 | 0 | 7 | 1.000 | 1.000 | No | Logical remainder |
| 10011 | 1 | 0 | 0 | 1 | 1 | 3 | 0.777 | 0.555 | No | Logical remainder |
| 10100 | 1 | 0 | 1 | 0 | 0 | 7 | 0.823 | 0.670 | No | Logical remainder |
| 10101 | 1 | 0 | 1 | 0 | 1 | 0 | - | - | No | Logical remainder |
| 10110 | 1 | 0 | 1 | 1 | 0 | 9 | 0.845 | 0.562 | No | Logical remainder |
| 10111 | 1 | 0 | 1 | 1 | 1 | 8 | 0.887 | 0.761 | No | Logical remainder |
| 11000 | 1 | 1 | 0 | 0 | 0 | 4 | 0.676 | 0.008 | No | Logical remainder |
| 11001 | 1 | 1 | 0 | 0 | 1 | 3 | 0.919 | 0.789 | No | Logical remainder |
| 11010 | 1 | 1 | 0 | 1 | 0 | 4 | 1.000 | 1.000 | No | Logical remainder |
| 11011 | 1 | 1 | 0 | 1 | 1 | 18 | 0.739 | 0.465 | Yes | 0 |
| 11100 | 1 | 1 | 1 | 0 | 0 | 9 | 0.858 | 0.700 | No | Logical remainder |
| 11101 | 1 | 1 | 1 | 0 | 1 | 6 | 0.934 | 0.822 | No | Logical remainder |
| 11110 | 1 | 1 | 1 | 1 | 0 | 16 | 0.949 | 0.918 | Yes | 1 |
| 11111 | 1 | 1 | 1 | 1 | 1 | 202 | 0.964 | 0.950 | Yes | 1 |

*Note: Frequency threshold = 10; raw consistency threshold = 0.85; PRI threshold = 0.70. The retained rows cover 79.1% of cases.*

**Supplementary Table S2 Complete Chinese-English item bank**

| Code | Chinese wording | English wording | Analytical status |
| --- | --- | --- | --- |
| Q1 | 岗位与县域服务需求匹配度 | Fit between the position and county-level service needs | Retained |
| Q2 | 基层诊疗服务工作成就感 | Sense of achievement in primary clinical service | Retained |
| Q3 | 县域医疗从业者的职业认可度 | Professional recognition of county-level health professionals | Retained |
| Q4 | 基层接诊压力与事务性工作负荷 | Clinical workload and administrative burden in primary care | Removed before final 31-item scoring |
| Q5 | 县域医院内部协作氛围 | Internal collaboration climate in county-level hospitals | Removed before final 31-item scoring |
| Q6 | 基层诊疗硬件设施完备度 | Adequacy of facilities for primary clinical care | Retained |
| Q7 | 县域医院后勤保障及时性 | Timeliness of logistical support in county-level hospitals | Retained |
| Q8 | 基层医护参与医院管理的便捷渠道 | Accessible channels for frontline staff participation in hospital management | Retained |
| Q9 | 县域团队专业技能互补性 | Complementarity of professional skills within county-level teams | Retained |
| Q10 | 县域医院诊疗和行政流程简化适配性 | Adaptation and streamlining of clinical and administrative processes | Retained |
| Q11 | 面向县域职工的制度执行公平性 | Fair implementation of institutional policies for county-level staff | Retained |
| Q12 | 贴合县域工作实际的考核指标科学性 | Validity of performance indicators adapted to county-level work | Retained |
| Q13 | 县域医院跨部门协作畅通度 | Effectiveness of interdepartmental collaboration in county-level hospitals | Retained |
| Q14 | 县域医疗岗位基本薪酬保障 | Adequacy of base compensation for county-level medical positions | Retained |
| Q15 | 基层诊疗工作量与薪酬匹配度 | Alignment between primary-care workload and compensation | Retained |
| Q16 | 县域医护带薪休假、轮休保障 | Provision of paid leave and rotational rest for county-level staff | Retained |
| Q17 | 针对县域人才的专项激励 | Targeted incentives for county-level professionals | Retained |
| Q18 | 县域医院内部晋升通道 | Internal promotion pathways in county-level hospitals | Retained |
| Q19 | 面向基层的实用技能培训 | Practical skills training for primary care | Retained |
| Q20 | 县域医护在职教育支持 | Support for continuing education of county-level staff | Retained |
| Q21 | 院部领导对县域医疗需求的把控与决策能力 | Hospital leaders’ understanding of and decision-making capacity for county-level health needs | Retained |
| Q22 | 科室主任业务指导能力与人文关怀 | Department heads’ professional guidance and humanistic support | Retained |
| Q23 | 职能科室领导服务临床一线的效率 | Efficiency of administrative departments in supporting frontline clinical services | Retained |
| Q24 | 县域医患沟通顺畅度 | Quality of clinician–patient communication in county-level settings | Retained |
| Q25 | 同事间工作关系融洽 | Positive working relationships among colleagues | Retained |
| Q26 | 上下级在医疗任务中的协作信任度 | Collaborative trust between supervisors and staff in clinical tasks | Retained |
| Q27 | 亲属、县域居民对医护职业的认可度 | Recognition of medical professions by family members and county residents | Retained |
| Q28 | 县域医院学术交流与基层科研支持 | Academic exchange and research support in county-level hospitals | Retained |
| Q29 | 县域特色专科建设水平 | Development of distinctive specialties in county-level hospitals | Retained |
| Q30 | 县级医院本土人才留用与培养机制 | Local workforce retention and development mechanisms in county-level hospitals | Retained |
| Q31 | 县域经济水平与医疗投入力度 | County economic development and health investment | Retained |
| Q32 | 医院在本县域及周边的竞争力 | Hospital competitiveness within the county and surrounding areas | Retained |
| Q33 | 与同级医院的薪酬福利和发展机会差距 | Gap in compensation, benefits, and development opportunities compared with peer hospitals | Retained |
| Y1 | 1.我常常想要辞去目前的工作 | I often think about quitting my present job. | Administered negative wording; deposited variable Y1_R_stored already transformed as 6 minus the original response; retained. |
| Y2 | 2.在明年可能会离开本单位另谋他就 | I may leave this organization for another job next year. | Administered negative wording; deposited variable Y2_R_stored already transformed as 6 minus the original response; retained. |
| Y3 | 3.计划在本单位长期发展职业 | I plan to develop my career in this organization over the long term. | Administered positive wording; deposited variable Y3_stored retained in its original polarity; not reverse-scored. |
| Y4 | 4.假如继续呆在本单位，前景不会好 | If I continue to stay in this organization, my prospects will not be good. | Administered negative wording; deposited variable Y4_R_stored already transformed as 6 minus the original response; retained. |
